# Supplementary material for: Quantitative comparison of a mobile, tablet-based eye-tracker and two stationary, video-based eye-trackers
Source: Behav Res Methods. 2025 Jan 6;57(1):45. doi: 10.3758/s13428-024-02542-w (PMC11703885; doi:10.3758/s13428-024-02542-w)
Supplement: Supplementary file 1 — Supplementary file1 (DOCX 22 KB) [file 13428_2024_2542_MOESM1_ESM.docx]

|  | ***Pro- & Anti-Saccades task: individual evaluation*** | | | | | | | | |
| --- | --- | --- | --- | --- | --- | --- | --- | --- | --- |
|  | **EL** | | | **TOM-rm** | | | **TOM-rs** | | |
|  | *mean  value* | *interi.  SD* | *interd.  SD* | *mean  value* | *interi.  SD* | *interd.  SD* | *mean  value* | *interi.  SD* | *interd.  SD* |
| *ER pro [%]* | 2.41 | 2.70 | 0.43 | 2.60 | 2.59 | 0.22 | 2.60 | 2.59 | 0.22 |
| *ER anti [%]* | 8.68 | 10.83 | 1.53 | 9.32 | 10.93 | 1.04 | 9.88 | 11.07 | 1.52 |
| *Num. sac.* | 105.81 | 36.13 | 3.64 | 100.14 | 36.21 | 5.75 | 105.05 | 37.35 | 3.63 |
| *Dur. of fix. [s]* | 0.80 | 0.11 | 0.03 | 0.80 | 0.08 | 0.05 | 0.80 | 0.10 | 0.04 |
| *Gain pro* | 1.00 | 0.06 | 0.02 | **0.93** | **0.06** | **0.03** | 0.98 | 0.06 | 0.03 |
| *Gain anti* | 0.99 | 0.08 | 0.03 | **0.92** | **0.08** | **0.02** | 0.97 | 0.08 | 0.03 |
| *Lat. pro [ms]* | 172.04 | 31.08 | 2.39 | **155.76** | **28.51** | **3.56** | 172.94 | 31.42 | 2.19 |
| *Lat. anti [ms]* | 215.32 | 43.07 | 2.97 | **197.09** | **40.23** | **4.95** | 216.67 | 42.69 | 2.52 |
|  | ***Pro- & Anti-Saccades task: same evaluation*** | | | | | | | | |
|  | **EL** | | | **TOM-rm** | | | **TOM-rs** | | |
|  | *mean  value* | *interi.  SD* | *interd.  SD* | *mean  value* | *interi.  SD* | *interd.  SD* | *mean  value* | *interi.  SD* | *interd.  SD* |
| *ER pro [%]* | 2.51 | 2.66 | 0.00 | 2.51 | 2.66 | 0.00 | 2.51 | 2.66 | 0.00 |
| *ER anti [%]* | 9.05 | 8.90 | 0.36 | 8.75 | 8.85 | 0.71 | 9.05 | 8.90 | 0.36 |
| *Num. sac.* | 108.71 | 38.19 | 4.07 | **102.76** | **38.30** | **5.75** | 110.67 | 39.37 | 7.12 |
| *Dur. of fix. [s]* | 0.93 | 0.14 | 0.03 | **0.80** | **0.08** | **0.05** | 0.92 | 0.13 | 0.03 |
| *Gain pro* | 1.00 | 0.05 | 0.02 | **0.93** | **0.06** | **0.02** | **0.97** | **0.06** | **0.02** |
| *Gain anti* | 0.99 | 0.08 | 0.02 | **0.92** | **0.07** | **0.02** | **0.96** | **0.08** | **0.02** |
| *Lat. pro [ms]* | 170.34 | 32.80 | 2.96 | **157.38** | **29.09** | **4.83** | **167.16** | **32.30** | **2.99** |
| *Lat. anti [ms]* | 210.74 | 42.36 | 3.19 | **197.29** | **39.64** | **4.44** | 209.04 | 41.27 | 1.97 |

**Table S 1:** Inter-individual mean values. Inter-individual SD (interi. SD) and inter-device SD (interd. SD) for eye movement parameters in the free viewing task. measured with EL, TOM-rm, and TOM-rs and analyzed with individual saccades and fixation detector (top) and with the same saccades and fixation detector (bottom). The boldly highlighted values differ significantly from the EL values.

|  | ***Free viewing task: individual evaluation*** | | | | | | | | |
| --- | --- | --- | --- | --- | --- | --- | --- | --- | --- |
|  | **EL** | | | **TOM-rm** | | | **TOM-rs** | | |
|  | *mean  value* | *interi.  SD* | *interd.  SD* | *mean  value* | *interi.  SD* | *interd. SD* | *mean*  *value* | *interi.*  *SD* | *interd.*  *SD* |
| *Dur. of fix. [s]* | 0.37 | 0.07 | 0.04 | 0.35 | 0.04 | 0.04 | 0.36 | 0.05 | 0.02 |
| *Num. of fix.* | 15.10 | 2.47 | 1.32 | **11.72** | **0.78** | **1.10** | **14.07** | **1.47** | **0.68** |
| *Sac. amp. [°]* | 3.64 | 0.50 | 0.12 | **3.09** | **0.38** | **0.19** | 3.57 | 0.60 | 0.22 |
| *time min.  area [s]* | 1.06 | 0.22 | 0.12 | - | - | - | 1.09 | 0.27 | 0.12 |
| *min. area*  *[a.u.]* | -4.50 | 0.37 | 0.25 | - | - | - | -4.42 | 0.59 | 0.25 |
| *light ref. [s]* | 224.38 | 47.91 | 17.62 | - | - | - | 232.19 | 56.85 | 17.62 |
| *light ref. [s]* | 423.81 | 112.99 | 82.90 | - | - | - | 419.62 | 135.92 | 82.90 |
|  | ***Free viewing task: same evaluation*** | | | | | | | | |
|  | **EL** | | | **TOM-rm** | | | **TOM-rs** | | |
|  | *mean  value* | *interi.  SD* | *interd.  SD* | *mean  value* | *interi.  SD* | *interd. SD* | *mean*  *value* | *interi.*  *SD* | *interd.*  *SD* |
| *Dur. of fix. [s]* | 0.53 | 0.11 | 0.04 | **0.35** | **0.05** | **0.06** | 0.53 | 0.11 | 0.04 |
| *Num. of fix.* | 11.07 | 1.64 | 0.49 | 11.72 | 0.78 | 0.76 | 10.81 | 1.46 | 0.47 |
| *Sac. amp. [°]* | 3.60 | 0.46 | 0.14 | **3.12** | **0.38** | **0.18** | 3.57 | 0.53 | 0.24 |

**Table S 2:** Inter-individual mean values, inter-individual SD (interi. SD) and inter-device SD (interd. SD) for eye movement parameters in the free viewing task. measured with EL, TOM-rm, and TOM-rs and analyzed with individual saccades and fixation detector (top) and with the same saccades and fixation detector (bottom). The boldly highlighted values differ significantly from the EL values.
